# Circulating PCSK9 Level and Risk of Cardiovascular Events and Death in Hemodialysis Patients

**DOI:** 10.3390/jcm9010244

**Published:** 2020-01-17

**Authors:** Hyeon Seok Hwang, Jin Sug Kim, Yang Gyun Kim, So-Young Lee, Shin Young Ahn, Hong Joo Lee, Dong-Young Lee, Sang Ho Lee, Ju Young Moon, Kyung Hwan Jeong

**Affiliations:** 1 Division of Nephrology, Department of Internal Medicine, Kyung Hee University, Seoul 02447, Korea; hwanghsne@gmail.com (H.S.H.); jinsuk0902@hanmail.net (J.S.K.); apple8840@hanmail.net (Y.G.K.); lshkidney@khu.ac.kr (S.H.L.); 2 Division of Nephrology, Department of Internal Medicine, CHA Bundang Medical Center, CHA University, Seongnam-si 13496, Korea; ysy0119@cha.ac.kr; 3 Division of Nephrology, Department of Internal Medicine, College of Medicine, Korea University, Seoul 08308, Korea; ahnshinyoung712@gmail.com; 4 Division of Nephrology, Department of Internal Medicine, Seoul Red Cross Hospital, Seoul 03181, Korea; moeru1@naver.com; 5 Division of Nephrology, Department of Internal Medicine, Veterans Health Service Medical Center, Seoul 05368, Korea; biizz@hanmail.net

**Keywords:** cardiovascular disease, hemodialysis, PCSK9

## Abstract

Proprotein convertase subtilisin/kexin type 9 (PCSK9) is a promising new target for the prevention of cardiovascular (CV) events. However, the clinical significance of circulating PCSK9 is unclear in hemodialysis (HD) patients. A total of 353 HD patients were prospectively enrolled from June 2016 to August 2019 in a K-cohort. Plasma PCSK9 level was measured at the time of study enrollment. The primary endpoint was defined as a composite of CV event and death. Plasma PCSK9 level was positively correlated with total cholesterol level in patients with statin treatment. Multivariate linear regression analysis revealed that baseline serum glucose, albumin, total cholesterol, and statin treatment were independent determinants of circulating PCSK9 levels. Cumulative rates of composite and CV events were significantly higher in patients with tertile 3 PCSK9 (*p* = 0.017 and *p* = 0.010, respectively). In multivariate Cox-regression analysis, PCSK9 tertile 3 was associated with a 1.97-fold risk of composite events (95% CI, 1.13–3.45), and it was associated with a 2.31-fold risk of CV events (95% CI, 1.17–4.59). In conclusion, a higher circulating PCSK9 level was independently associated with incident CV events and death in HD patients. These results suggest the importance of future studies regarding the effect of PCSK9 inhibition.

## 1. Introduction

Patients receiving hemodialysis (HD) have an increased risk of cardiovascular disease [1,2]. Low-density lipoprotein (LDL) cholesterol is one of the most well-established and strongest risk factors for cardiovascular (CV) events in the general population, and it remains associated with CV events in patients with non-dialysis chronic kidney disease [3]. However, the association between higher LDL and CV risk is weaker for patients with lower renal function, and LDL cholesterol level has an inconsistent association with all-cause mortality in patients on dialysis treatment [4,5]. In addition, statin treatment to reduce LDL failed to prevent CV events in HD patients, and kidney disease: improving global outcomes (KDIGO) guidelines recommend that statins should not be initiated [6,7,8].

Hepatic extracellular LDL receptors uptake LDL cholesterol, which acts as the main mechanism for LDL clearance [9]. Proprotein convertase subtilisin/kexin type 9 (PCSK9) inhibits the recycling of LDL receptors and promotes their degradation [10,11]. Low levels of circulating PCSK9 ultimately reduce plasma LDL, and PCSK9 has emerged as a major regulator of LDL cholesterol. Circulating PCSK9 levels have previously been shown to correlate with coronary artery calcification and are associated with risk of CV events [12,13]. It has recently been suggested that PCSK9 level is correlated with LDL and total cholesterol level in patients with nephrotic syndrome, chronic kidney disease, or dialysis treatment [14,15]. In addition, circulating PCSK9 level is dependent on different renal replacement modality [16,17]. However, PCSK9 level is rarely evaluated in patients receiving HD treatment, and there are no reports investigating the prognostic significance of PCSK9 in patients receiving dialysis treatment.

Therefore, we undertook this study to test the hypothesis that circulating PCSK9 level is independently associated with an increased risk of future CV events and death in HD patients. We also explored serum lipid levels and clinical parameters to determine possible correlations with PCSK9 level.

## 2. Materials and Methods

### 2.1. Study Population

All patients enrolled in this study were on the registry of the K-cohort. The K-cohort is a multicenter, internet-based, prospective cohort of HD patients in Korea that was designed to investigate prognostic markers for CV complications and death and to improve survival rates and quality of life. Enrollment commenced in June 2016 and included adult (>18 years of age) HD patients from 6 general hospitals (Kyung Hee University Medical Center, Kyung Hee University Hospital at Gangdong, CHA Bundang Medical Center, Korea University Guro Hospital, Seoul Red Cross Hospital, Veterans Health Service Medical Center). The inclusion criteria for the K-cohort were regular 4 h HD prescriptions per session occurring 3 times a week for at least 3 months. The exclusion criteria were pregnancy, hematologic malignancy, active or invasive solid tumor, and less than 6 months of life expectancy. A total of 452 patients were screened from June 2016 to August 2019, and a final 353 patients with whole blood, serum, and plasma samples at the time of study enrollment were enrolled.

The study protocol was approved by the local Ethics Committee (KHNMC 2016-04-039), and the study was conducted in accordance with the principles of the second Declaration of Helsinki. All participants involved in the study signed written informed consent forms before enrollment.

### 2.2. Data Collection and Definitions

Demographic factors, relevant medical history, comorbid conditions, concomitant medication, laboratory data, and dialysis information were ascertained at the time of inclusion by reviewing medical records and patient interviews. Information on comorbidities that constitutes the Charlson comorbidity index was derived and used to calculate the index score [18]. Laboratory data were collected from fasting blood samples before the start of HD in a midweek session; hemoglobin, serum glucose and albumin, BUN, creatinine, total cholesterol, triglycerides, LDL cholesterol, high-sensitivity C-reactive peptide (hsCRP), and intact-parathyroid hormone (i-PTH) levels were measured. Delivered *spKt/V* (K, dialyzer clearance; t, time; V, urea distribution volume) was assessed using the conventional method [19], and body mass index (BMI) was defined as body weight divided by the square of body height.

Patients were classified into three groups based on PCSK9 distribution: tertile 1, <26.5 ng/mL, tertile 2, 26.5–41.5 ng/mL, tertile 3, ≥41.5 ng/mL. All patients were followed up prospectively after all baseline assessments. Clinical events were identified, including all-cause mortality and CV events. Patient follow-up was censored at the time of transfer to peritoneal dialysis, kidney transplantation, follow-up loss, or patient withdrawal.

### 2.3. Laboratory Measurements

Plasma samples for measurement of monocyte chemoattractant protein (MCP)-1, interleukin (IL)-6, osteoprotegerin, receptor activator of nuclear factor kappa-Β ligand (RANKL), and PCSK9 were collected using ethylenediaminetetraacetic acid-treated tubes at the time of study entry. After centrifugation for 15 min at 1000× *g* at room temperature, samples were stored at −80 °C until use. The enzyme-linked immunosorbent assay method was performed using Magnetic Luminex^®^ Screening Assay multiplex kits (R&D Systems, Inc., Minneapolis, MN, USA).

### 2.4. Outcome Measures

The primary study endpoint was a composite of incident CV events and death. CV events were defined as coronary artery disease (coronary artery bypass surgery, percutaneous intervention, or myocardial infarction), heart failure, ventricular arrhythmia, cardiac arrest, cerebral infarction, and peripheral vascular occlusive diseases requiring revascularization or surgical intervention. All mortality events from any cause were retrieved and carefully reviewed. Secondary endpoints were clinical and laboratory parameters, which were correlated with PCSK9 level.

### 2.5. Statistical Analysis

Data are expressed as mean ± standard deviation. Differences between the three groups were identified using ANOVA. Categorical variables were compared using the chi-square test or Fisher’s exact test. Log-transformed values of hs-CRP were used in regression analysis because of a skewed distribution. Spearman’s analyses were used to evaluate the correlation between PCSK9 and continuous variables. The association between PCSK9 level and relevant factors was identified using linear regression analysis. Established clinical and metabolic risk factors that were associated with plasma PCSK9 concentrations in published reports were also included [16,20]. The cumulative event rates were estimated by the Kaplan–Meier method and compared using the log-rank test. The Cox proportional-hazards model was constructed to identify independent variables related to patient death or CV event. Multivariate models included significantly associated parameters according to their weight in univariate testing and clinically fundamental parameters. The confounders entered into analysis were age, sex, BMI, Charlson comorbidity index, hemoglobin concentration, serum glucose and albumin level, total cholesterol, LDL cholesterol, statin use, dialysis duration, *spKt/V*, and catheter as vascular access. Formal tests for interaction between PCSK9 and predefined subgroups were conducted in addition to the main effects of the fully adjusted models. We modeled the association between PCSK9 and the probability to develop CV event and death. We chose to use knots located at each of the PCSK9 tertile cutoff values plus knots at the minimum and maximum values. Restricted cubic spline transformations were applied to continuous measures. The sample size was estimated that would be needed for the composite of CV event and death (with α error = 0.05; β error = 0.20; hazard ratio = 1.75). The required number of patients was 98 in each group. *p* values <0.05 were considered significant. Statistical analyses were performed using SPSS software (version 20.0; SPSS, IBM Corp., Armonk, NY, USA) and R software (version R 3.6.2; https://cran.r-project.org/).

## 3. Results

### 3.1. Baseline Demographic Characteristics and Laboratory Data

The mean concentration of PCSK9 level was 36.6 ± 20.3 ng/mL in all studied patients. Mean PCSK9 concentration was 17.8 ± 5.5 ng/mL in tertile 1 (*n* = 118), 33.6 ± 4.1 ng/mL in tertile 2 (*n* = 117), and 58.3 ± 18.6 ng/mL in tertile 3 (*n* = 118).

Baseline patient demographics, clinical characteristics, and laboratory results are presented in Table 1. Patients with tertile 3 PCSK9 were more often diabetic, had shorter duration dialysis therapy, and more frequent statin use than those with lower PCSK9 level. Serum albumin level was significantly lower and serum glucose and triglyceride level was higher in patients with tertile 3 PCSK9 than in those with tertile 1 or 2 PCSK9. Total and LDL cholesterol level and dialysis characteristics did not differ across tertiles.

### 3.2. Determinant Factors for Circulating PCSK9 Level in HD Patients

The correlations between PCSK9 level and lipid parameters were evaluated according to statin treatment (Table S1). In patients without statin treatment, no lipid parameters were associated with PCSK9 levels. Total cholesterol level was positively associated with PCSK9 level in patients with statin treatment (*ρ* = 0.154; *p* = 0.049). However, triglyceride, LDL cholesterol, and HDL cholesterol levels were not correlated. To investigate the possible relationship of PCSK9 with inflammation and vascular calcification, the correlation between PCSK9 level and correspondent cytokines was also evaluated. Serum hsCRP, plasma MCP-1, and plasma IL-6 were used as markers for inflammation, and osteoprogegerin and RANKL were considered relevant factors for calcification. However, no inflammatory cytokines or calcification-related factors showed significant correlation with plasma PCSK9 level.

Baseline clinical and laboratory determinants for plasma PCSK9 level were evaluated. In univariate analysis, duration of receiving HD and serum albumin level were negatively correlated with PCSK9 level. The age, sex, BMI and hemoglobin level did not have significant correlation with PCSK9 level. Serum glucose and triglyceride levels and statin use were positive determinants for PCSK9 level. In multivariate analysis, serum glucose, serum albumin, total cholesterol, and use of statin were independent determinants of PCSK9 level (Table 2).

### 3.3. Risk of CV Events and Death in Different PCSK9 Level

**Figure 1:**
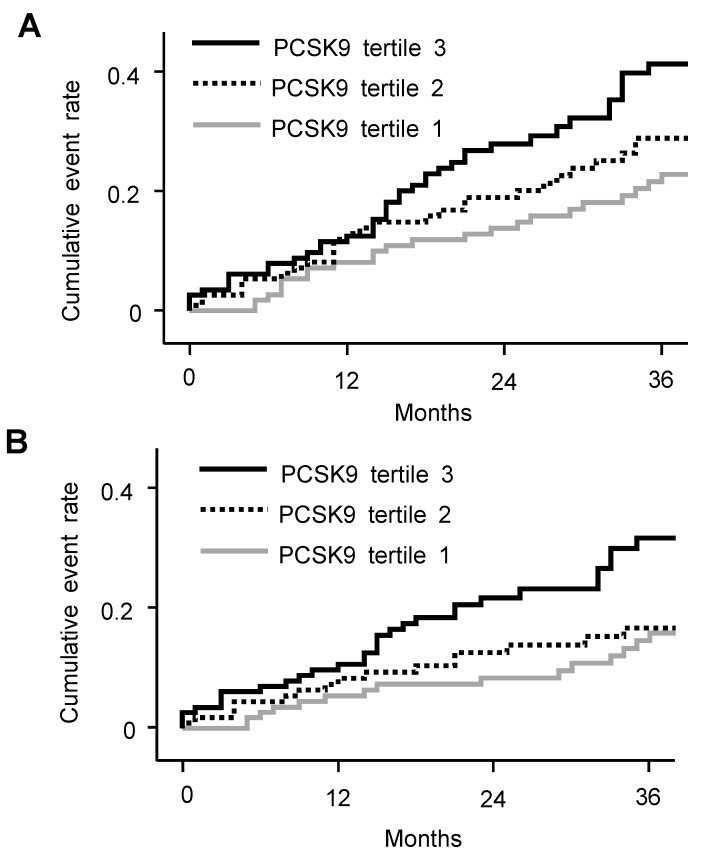
Cumulative event rates for composite of CV events and death (**A**) and CV events (**B**) according to the PCSK9 level.

During follow-up, 30 deaths (8.5%) and 60 CV events (17.0%) occurred. The cumulative event rate for composite of CV events and death was significantly greater as PCSK9 level increased (*p* = 0.017; Figure 1). PCSK9 tertile 3 was associated with higher cumulative event rate of CV events (*p* = 0.010). The cumulative event rate of death did not differ between patients with different PCSK9 levels (*p* = 0.217).

We compared the type and incidence of CV events across tertiles. The incidence of all CV events and stroke was significantly greater in patients with tertile 3 PCSK9. The incidence of coronary artery disease, heart failure, and peripheral artery disease was higher in tertile 3 PCSK9, but it was not statistically significant (Table 3).

Univariable Cox-regression revealed a significant association between plasma tertile 3 PCSK9 and composite events (hazard ratios [HR], 2.05; 95% confidence interval [CI], 1.01–3.09; *p* = 0.007; Table 4). This association remained significant after adjustment for multiple variables (HR, 1.97; 95% CI, 1.13–3.45; *p* = 0.017). Plasma PCSK9 increment per 1 ng/mL revealed a significant association with increased risk of composite events (HR, 1.02; 95% CI, 1.004–1.026; *p* = 0.009). The observed HR for CV events and patient death were evaluated, respectively. Patients with tertile 3 PCSK9 had an independent risk of CV events after multiple adjustment (HR, 2.31; 95% CI, 1.17–4.59; *p* = 0.017). Tertile 3 PCSK9 showed 1.45-fold increased risk of patient death without statistical significance (95% CI, 0.65–3.27; *p* = 0.367).

**Figure 2:**
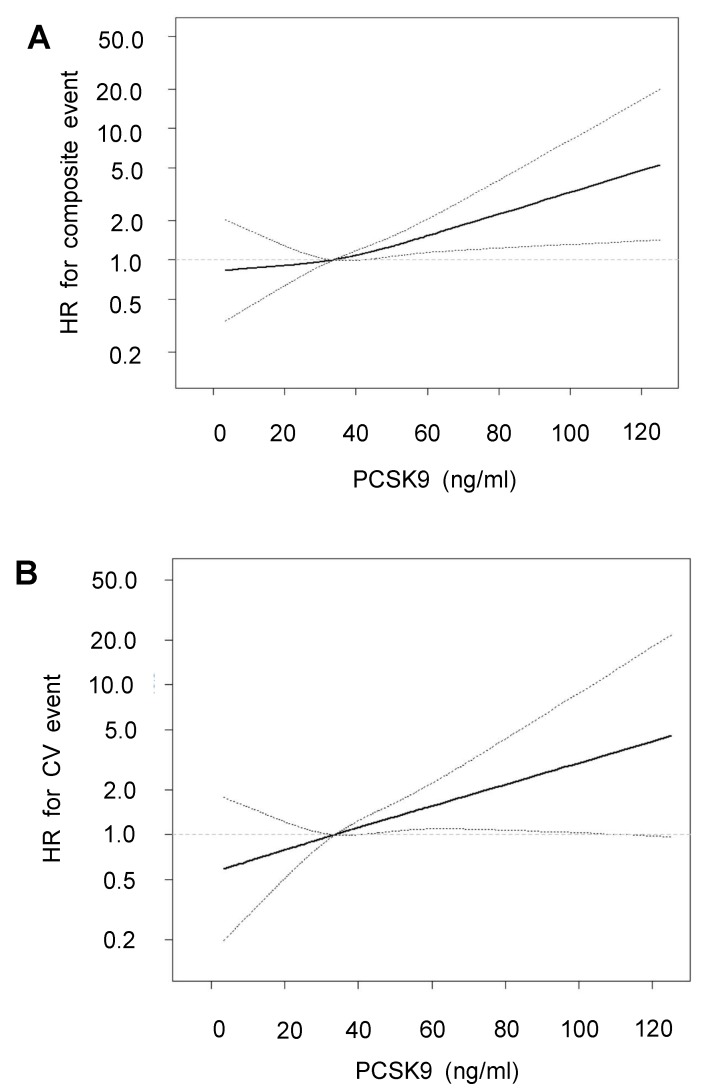
Linear associations of PCSK9 and risk of composite (**A**) and of CV event (**B**) after multiple adjustments. Dashed lines represent 95% confidence intervals. The adjusted multiple variables were age, sex, BMI, Charlson comorbidity index, dialysis duration, *spKt/V*, catheter use, hemoglobin, serum glucose and albumin, log hsCRP, statin use, total cholesterol, and LDL cholesterol.

To evaluate potential linear associations, we evaluated the association between PCSK9 and the risk of composite and CV events during the follow-up period. The cubic restricted spline model after multiple adjustments shows gradually increasing HRs for composite and CV events with increasing PCSK9 (Figure 2).

The relationship between tertile 3 PCSK9 and incident composite events was further investigated in subgroups stratified by LDL cholesterol, and hsCRP level (Table 5). High PCSK9 was defined as tertile 3, and patients with tertile 1 and 2 PCSK9 were used as the reference category. Criteria for predefined subgroups were based on median values; high hsCRP, >0.85 mg/dL; high LDL > 75 mg/dL. We compared the cumulative event rate of the primary endpoint. The highest cumulative event rate of composite event was observed in patients with high PCSK9 and elevated LDL cholesterol (*p* = 0.028; Supplementary Materials Figure S1). When patients were classified based on hsCRP level, patients with a high PCSK9 level showed a higher cumulative event rate whether hsCRP was elevated or not (*p* = 0.039). Univariable Cox-regression revealed a similar association between a predefined subgroup and composite events. In multivariate Cox-regression model, elevated PCSK9 in the absence of an elevated LDL cholesterol level was not associated with the risk of a composite event (Table 5). However, high PCSK9 in combination with elevated LDL cholesterol showed a greater risk (HR, 2.59; 95% CI, 1.30–5.14; *p* = 0.007). A significant association between high PCSK9 and composite events was observed in patients with low hsCRP level (HR, 2.03; 95% CI, 1.06–3.89; *p* = 0.033), but high PCSK9 level was not a significant risk factor in patients with high hsCRP level.

## 4. Discussion

Our prospective observational study demonstrated that serum glucose, albumin, total cholesterol level, and statin treatment were independent determinants of PCSK9 level in HD patients. Patients with tertile 3 PCSK9 had greater risk of incident composite of CV events and death. This relationship persisted after adjustment for established cardiovascular risk factors and lipid parameters. In addition, higher level of PCSK9 provided additional risk in patients with increased level of LDL. These findings suggest that PCSK9 may be a novel biomarker for CV events in HD patients.

Lipid parameters and statin treatment have excellent correlation with PCSK9 levels in patients without renal dysfunction [21,22,23,24,25]. Consistently, a similar relationship was observed in patients with non-dialysis dependent chronic kidney disease (CKD) [14,15,16]. While total cholesterol level and statin treatment were independent determinants of PCSK9 level in this study, no significant relationship was identified between PCSK9 and triglyceride, LDL and HDL cholesterol. These findings suggest that the relationship of PCSK9 with lipid parameters becomes weaker in HD patients and that it does not exactly resemble that of the general population or patients with non-dialysis dependent CKD. We presumed that HD-induced dysregulation in lipid parameters and the effect of HD treatment on PCSK9 level reduced the strength of this relationship [8,16,20,26].

The pro-inflammatory effect of PCSK9 has been shown in different experimental models [27,28,29]. The inhibitory effect of PCSK9 on vascular calcification was also suggested in several basic experiments [30,31]. In addition, clinical evidence supported a linkage between PCSK9 and chemokine and vascular calcification in previous studies [24,32]. However, we did not find a significant correlation between PCSK9 level and hsCRP, MCP-1, or IL-6. In addition, the observed correlation of PCSK9 with osteoprotegerin and RANKL was not significant. These findings suggest that PCSK9 has limited association with systemic inflammation or calcification in HD patients and that the expected role of PCSK9 in this field might be reduced in HD patients.

While HD patients had the greatest risk of CV event and death, traditional risk factors such as hypertension, high LDL cholesterol levels, low HDL cholesterol levels, and smoking do not fully explain the elevated CV risk of HD patients. Our study revealed a significant association between plasma PCSK9 and incident composite and CV event in univariate analysis. The association remained significant after adjustment for multiple established CV risk factors, including lipid parameters and statin use. These findings suggest that PCSK9 contributes CV event independently of traditional CV risk factor and that PCSK9 is a useful biomarker for elucidating CV risk in patients on HD treatment.

Our study showed that the association between PCSK9 and composite event was insignificant in a subgroup with low LDL cholesterol. These findings suggest that the pleiotropic effect of PCSK9 beyond lipid metabolism is decreased in HD patients. In contrast, the combination of high PCSK9 with high LDL cholesterol showed the greatest risk of composite events. We propose that PCSK9 could be a marker used to screen high risk patients for CV events in combination with LDL cholesterol. In patients with high hsCRP, high PCSK9 was not a significant risk factor, but the greatest CV risk was observed in patients with high PCSK9 and low hsCRP. These findings suggest that the combination of high PCSK9 with high hsCRP indicates antagonistic effect on prognostic value of PCSK9.

This study has some limitations. Due to a limited number of CVD events and short-term follow-up duration, we could not perform individual analyses for myocardial infarction and stroke. Furthermore, control for confounding factors may not have considered all relevant factors such as atrial fibrillation and cannot preclude residual confounding in our data. In addition, PCSK9 level was measured once at the time of study entry, and information about statin treatment was assessed at baseline.

In conclusion, circulating PCSK9 level was independently correlated with serum glucose, albumin, total cholesterol level, and statin treatment. Higher circulating PCSK9 level was independently associated with greater risk of composites of CV event and death in HD patients. Our study suggests the importance of future studies on the effect of PCSK9 inhibition in HD patients.

## Figures and Tables

**Table 1:** Baseline demographic and laboratory data of study population.

	Circulating PCSK9 Level			*p*
	Tertile 1 (*n* = 118)	Tertile 2 (*n* = 117)	Tertile 3 (*n* = 118)	
Age (years)	62.1 ± 12.4	61.7 ± 13.7	62.8 ± 12.2	0.791
Male (%)	78 (66.1)	80 (68.4)	79 (66.9)	0.932
Body mass index (kg/m^2^)	23.0 ± 3.7	22.6 ± 3.5	23.7 ± 4.5	0.079
HD duration (months)	70.0 ± 76.7	55.4 ± 53.8	47.8 ± 60.7 ^a^	0.029
Diabetes (%)	57 (48.3)	60 (51.3)	79 (66.9) ^a,b^	0.008
History of CVE (%)	52 (44.1)	46 (39.3)	60 (50.8)	0.203
Charlson comorbidity score	3.9 ± 1.6	3.9 ± 1.5	4.3 ± 1.4	0.055
Follow-up duration (months)	30.9 ± 10.6	27.9 ± 12.7	28.0 ± 10.9	0.072
Statin use (%)	33 (28.0)	55 (47.0) ^a^	76 (64.4) ^a,b^	<0.001
PCSK9 (ng/mL)	17.8 ± 5.5	33.6 ± 4.1 ^a^	58.3 ± 18.6 ^a,b^	<0.001
Hemoglobin (g/dL)	10.5 ± 1.0	10.3 ± 1.2	10.4 ± 1.4	0.396
Serum glucose	142.6 ± 51.3	146.8 ± 54.3	171.4 ± 75.6 ^a,b^	0.001
Albumin (g/dL)	4.0 ± 0.3	3.9 ± 0.4 ^a^	3.8 ± 0.4 ^a,b^	0.017
Total cholesterol (mg/dL)	134.5 ± 29.0	135.3 ± 30.3	137.9 ± 31.6	0.668
Triglyceride (mg/dL)	117.4 ± 78.7	108.3 ± 68.8	134.6 ± 80.4 ^b^	0.028
LDL-cholesterol (mg/dL)	77.1 ± 26.3	77.1 ± 24.8	77.1 ± 27.4	1.00
HDL-cholesterol (mg/dL)	45.5 ± 13.9	45.2 ± 13.2	43.4 ± 11.4	0.420
hsCRP (mg/dL)	3.5 ± 6.7	4.7 ± 9.7	3.5 ± 7.5	0.424
i-PTH	288.0 ± 252.3	280.8 ± 210.8	246.9 ± 193.3	0.312
Predialysis SBP (mmHg)	143.0 ± 20.3	143.0 ± 19.5	144.3 ± 21.0	0.855
UF (L)	2.2 ± 1.1	2.3 ± 1.1	2.2 ± 1.1	0.804
spKt/V	1.57 ± 0.29	1.56 ± 0.30	1.56 ± 0.31	0.967
Catheter use (%)	1 (0.8)	5 (4.3)	8 (6.8)	0.066

CVE, cardiovascular event; HD, hemodialysis; HDL-cholesterol, high-density lipoprotein cholesterol; hsCRP, high-sensitivity C-reactive protein; LDL-cholesterol, low-density lipoprotein cholesterol; PTH, parathyroid hormone; SBP, systolic blood pressure; UF, ultrafiltration. ^a^ *p* < 0.05 vs. tertile 1; ^b^ *p* < 0.05 vs. tertile 2.

**Table 2:** Determinants for PCSK9 level in HD patients.

	*Unstandardized β*	95% CI	*p*
HD duration (months)	−0.011	−0.043, 0.021	0.507
Glucose (mg/dL)	0.047	0.014, 0.080	0.005
Albumin (g/dL)	−8.292	−14.477, −2.107	0.009
Total cholesterol (mg/dL)	0.134	0.023, 0.244	0.018
Triglyceride (mg/dL)	0.018	−0.011, 0.047	0.216
LDL-cholesterol (mg/dL)	−0.093	−0.217, 0.031	0.141
i-PTH (pg/mL)	−0.004	−0.013, 0.006	0.435
Statin use	11.192	7.115, 15.269	<0.001

HD, hemodialysis; LDL-cholesterol, low-density lipoprotein cholesterol; PTH, parathyroid hormone.

**Table 3:** Incidence of CV events based on plasma PCSK9 level.

	Circulating PCSK9 Level			*p*
	Tertile 1	Tertile 2	Tertile 3	
**All CV events (%)**	15 (12.7)	16 (13.7)	29 (24.6)	0.027
Coronary artery disease (%)	9 (7.6)	9 (7.7)	14 (11.9)	0.431
Heart failure (%)	3 (2.5)	2 (1.7)	4 (3.4)	0.912
Stroke (%)	1 (0.8)	0	6 (5.1)	0.019
Peripheral artery disease (%)	0	1 (0.9)	4 (3.4)	0.092
Cardiac arrest (%)	2 (1.7)	4 (3.4)	1 (0.8)	0.320

CV, cardiovascular.

**Table 4:** Hazard ratios of plasma PCSK9 tertiles for CV events and death.

	No. of Events (%)	HR (95% CI), Crude	HR (95% CI), Adjusted ^a^
**Composite events**			
PCSK9 tertile 1	23 (19.5)	Reference	Reference
PCSK9 tertile 2	28 (23.9)	1.35 (0.78–2.35)	1.33 (0.75–2.36)
PCSK9 tertile 3	39 (33.1)	2.05 (1.22–3.43)	1.97 (1.13–3.45)
PCSK9 increase per 1 ng/mL		1.01 (1.001–1.019)	1.02 (1.004–1.026)
**CV events**			
PCSK9 tertile 1	15 (12.7)	Reference	Reference
PCSK9 tertile 2	16 (13.7)	1.18 (0.58–2.39)	1.28 (0.61–2.66)
PCSK9 tertile 3	29 (24.6)	2.31 (1.24–4.32)	2.31 (1.17–4.59)
PCSK9 increase per 1 ng/mL		1.01 (1.003–1.024)	1.02 (1.004–1.030)
**Death**			
PCSK9 tertile 1	12 (10.2)	Reference	Reference
PCSK9 tertile 2	20 (17.1)	1.88 (0.92–3.84)	1.79 (0.85–3.78)
PCSK9 tertile 3	16 (13.6)	1.48 (0.70–3.14)	1.45 (0.65–3.27)
PCSK9 increase per 1 ng/mL		1.01 (0.993–1.019)	1.01 (0.997–1.032)

CV, cardiovascular; HR, hazard ratio; No., number. ^a^ All analyses are adjusted for the following covariates: age, sex, BMI, Charlson comorbidity index, dialysis duration, *spKt/V*, catheter use, hemoglobin, serum glucose and albumin, log hsCRP, statin use, total cholesterol, and LDL cholesterol.

**Table 5:** Hazard ratios of plasma tertile 3 PCSK9 for composite events based on predefined subgroup.

	No. of Events/No. of Patients	HR (95% CI), Crude	HR (95% CI), Adjusted ^a^	*p* for Interaction
**LDL-cholesterol**				0.370
Low LDL and low PCSK9	28/114 (24.6)	Reference	Reference	
Low LDL and high PCSK9	18/61 (29.5)	1.36 (0.75–2.46)	1.30 (0.69–2.46)	
High LDL and low PCSK9	23/121 (19.0)	0.83 (0.48–1.44)	1.16 (0.59–2.27)	
High LDL and high PCSK9	21/57 (36.8)	1.90 (1.08–3.36)	2.59 (1.30–5.14)	
**hsCRP**				0.443
Low hsCRP and low PCSK9	23/124 (18.5)	Reference	Reference	
Low hsCRP and high PCSK9	19/54 (35.2)	2.18 (1.19–4.00)	2.03 (1.06–3.89)	
High hsCRP and low PCSK9	28/111 (25.2)	1.38 (0.79–2.39)	1.09 (0.60–1.99)	
High hsCRP and high PCSK9	20/64 (31.2)	1.97 (1.08–3.58)	1.56 (0.81–3.02)	

HR, hazard ratio; hsCRP, high-sensitivity C-reactive protein; LDL-cholesterol, low-density lipoprotein cholesterol; No., number. High PCSK9 was defined as tertile 3, and criteria for predefined subgroups was based on median values; high LDL > 75 mg/dL; high hsCRP, >0.85 mg/dL. ^a^ All analyses are adjusted for the following covariates (except for the variable used to define the subgroup in each case): age, sex, BMI, Charlson comorbidity index, dialysis duration, *spKt/V*, catheter use, hemoglobin, serum glucose and albumin, log hsCRP, statin use, total cholesterol, and LDL cholesterol.

## Supplementary Materials

The following are available online at https://www.mdpi.com/2077-0383/9/1/244/s1, Figure S1: Cumulative event rates of plasma tertile 3 PCSK9, Table S1: Correlation of PCSK9 level with serum lipid, cytokines and bone-related protein levels.
