# Supplementary material for: Circulating PCSK9 Level and Risk of Cardiovascular Events and Death in Hemodialysis Patients
Source: J Clin Med. 2020 Jan 17;9(1):244. doi: 10.3390/jcm9010244 (PMC7019341; doi:10.3390/jcm9010244)
Supplement: Supplementary file 1 [file jcm-09-00244-s001.pdf]

Supplementary Materials:

Table S1. Correlation of PCSK9 level with serum lipid, cytokines and bone-related protein levels

	**No statin (n = 189)**	**Statin (n = 164)**
Total cholesterol (mg/dL)	0.071	0.154[a]
Triglyceride (mg/dL)	0.060	0.142
LDL (mg/dL)	0.049	0.072
HDL (mg/dL)	-0.023	-0.042
hsCRP (mg/dL)	0.034	-0.051
MCP-1 (pg/mL)	0.080	0.045
IL-6 (pg/ml)	0.092	0.043
Osteoprotegerin (pg/ml)	0.129	-0.039
RANKL (pg/ml)	0.094	-0.065

HD, hemodialysis; HDL, high-density lipoprotein cholesterol; hsCRP, high-sensitivity C-reactive protein; IL-6, interleukin-6; LDL, low-density lipoprotein cholesterol; MCP-1, monocyte chemoattractant protein-1; RANKL, receptor activator of nuclear factor kappa-B ligand. [a] $p < 0.05$

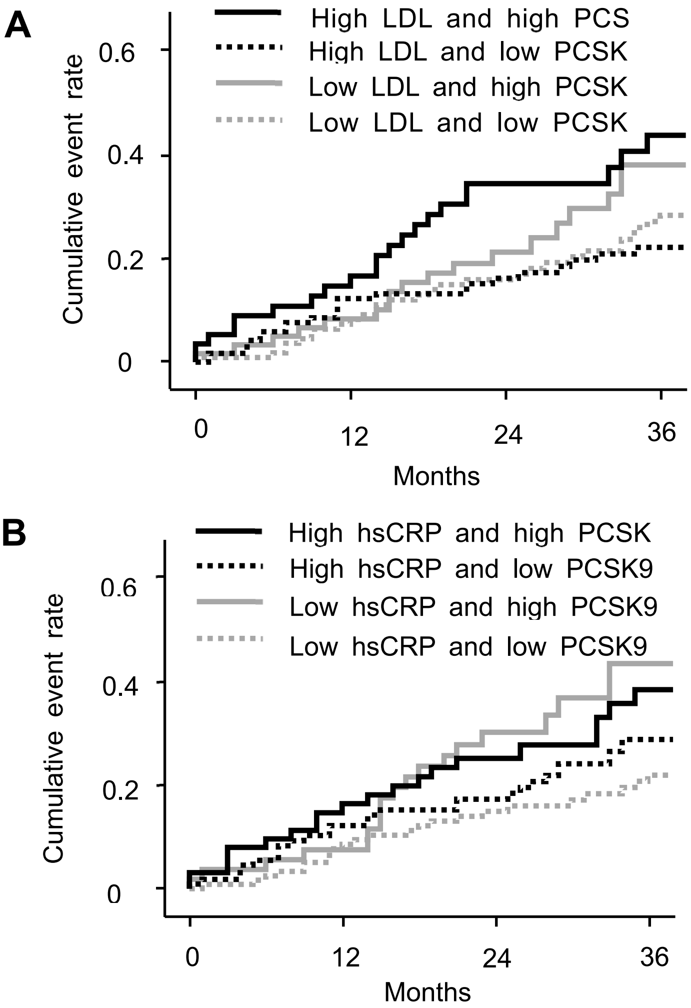

Figure S1. Cumulative event rates of plasma tertile 3 PCSK9 for composite events based on predefined LDL (A), and hsCRP level (B). High PCSK9 was defined as tertile 3, and criteria for predefined subgroups was based on median values; high LDL > 75 mg/dL; high hsCRP, > 0.85 mg/dL. hsCRP, high-sensitivity C-reactive protein; LDL-cholesterol, low-density lipoprotein cholesterol.
